# Current knowledge and recent insights into the genetic basis of amyotrophic lateral sclerosis

**DOI:** 10.1007/s11825-018-0185-3

**Published:** 2018-07-13

**Authors:** Alexander E. Volk, Jochen H. Weishaupt, Peter M. Andersen, Albert C. Ludolph, Christian Kubisch

**Affiliations:** 1 Institute of Human Genetics, University Medical Center Hamburg-Eppendorf, Martinistr. 52, 20246 Hamburg, Germany; 2 Department of Neurology, Ulm University, Ulm, Germany; 3 Department of Pharmacology and Clinical Neuroscience, Umeå University, Umeå, Sweden

**Keywords:** Motor neuron disease, Genetic heterogeneity, C9orf72, Oligogenic inheritance, Predictive testing, Motoneuronerkrankung, Genetische Heterogenität, C9orf72, Doppelmutationsträger, Prädiktive Testung

## Abstract

Amyotrophic lateral sclerosis (ALS) is the most frequent motor neuron disease, affecting the upper and/or lower motor neurons. However, extramotor symptoms can also occur; cognitive deficits are present in more than 40% of patients and 5–8% of ALS patients develop frontotemporal dementia. There is no effective treatment for ALS and median survival is 2–3 years after onset.

Amyotrophic lateral sclerosis is a genetically heterogeneous disorder with monogenic forms as well as complex genetic etiology. Currently, complex genetic risk factors are of minor interest for routine diagnostic testing or counseling of patients and their families. By contrast, a monogenic cause can be identified in 70% of familial and 10% of sporadic ALS cases. The most frequent genetic cause is a noncoding hexanucleotide repeat expansion in the *C9orf72* gene. In recent years, high-throughput sequencing technologies have helped to identify additional monogenic and complex risk factors of ALS.

Genetic counseling should be offered to all ALS patients and their first- and possibly second-degree relatives, and should include information about the possibilities and limitations of genetic testing. Routine diagnostic testing should at least encompass the most frequently mutated disease genes (*C9orf72*, *SOD1*, *TDP-43*, *FUS*). Targeted sequencing approaches including further disease genes may be applied. Caution is warranted as the *C9orf72* repeat expansion cannot be detected by routine sequencing technologies and testing by polymerase chain reaction (PCR) is failure-prone.

Predictive testing is possible in families in which a genetic cause has been identified, but the limitations of genetic testing (i. e., the problems of incomplete penetrance, variable expressivity and possible oligogenic inheritance) have to be explained to the families.

## Zusammenfassung

Die amyotrophe Lateralsklerose (ALS) ist die häufigste neurodegenerative Erkrankung des motorischen Nervensystems. Ursächlich ist der Untergang des ersten und/oder zweiten Motoneurons, wobei weitere neuronale Strukturen betroffen sein können. Neben motorischen Befunden können z. B. kognitive Defizite bestehen (mehr als 40 % der Patienten); eine frontotemporale Demenz entwickelt sich bei 5–8 % aller Patienten. Eine ursächliche Therapie gibt es derzeit nicht, die mittlere Überlebenszeit beträgt 2–3 Jahre.

Die ALS ist eine genetisch heterogene Erkrankung mit monogenen Formen und komplex-genetischen Risikofaktoren. Letztere spielen für diagnostische Testungen und bei Beratungen von ALS-Patienten und ihren Familien z. Zt. (noch) eine untergeordnete Rolle. Bei 70 % der familiären und 10 % der sporadischen ALS-Patienten können genetische Untersuchungen dagegen die Diagnose einer monogenen ALS sichern. Eine nichtkodierende Hexanukleotidrepeat-Expansion des *C9orf72*-Gens ist dabei die häufigste genetische Ursache einer ALS. Hochdurchsatzsequenzierungen haben in den letzten Jahren wesentlich dazu beigetragen, weitere genetische Risikofaktoren der ALS zu identifizieren.

ALS-Patienten sowie erst- und ggf. zweitgradig Verwandten sollte eine genetische Beratung angeboten werden, in der die Möglichkeiten und Limitationen von genetischen Testungen erläutert werden. Eine routinediagnostische Testung sollte zumindest die häufigsten Krankheitsgene (*C9orf72*, *SOD1*, *TDP-43*, *FUS*) umfassen. Panelanalysen mit weiteren Genen können durchgeführt werden. Einschränkend ist zu erwähnen, dass die Repeatexpansion des *C9orf72*-Gens durch Sequenzierung in der Regel nicht nachweisbar ist und auch PCR-basierte Analyseverfahren fehleranfällig sind.

Eine gezielte prädiktive Testung ist im Falle eines Mutationsnachweises möglich, wobei die Grenzen der Aussagekraft des genetischen Tests (inkomplette und altersabhängige Penetranz, variable Expressivität, eventuelles Vorliegen mehrerer pathogener Mutationen bei einer Person) verdeutlicht werden müssen.

## Introduction and clinical aspects

Amyotrophic lateral sclerosis (ALS) is a neurodegenerative disease that primarily affects the upper and lower motor neurons. The degeneration of the lower motor neurons results in the denervation of muscles followed by fasciculation, cramps, muscle wasting, and weakness. The degeneration of the upper motor neurons results in a loss of fine motor control of the lower motor neuron system, causing spastic paresis. The initial presentation varies between patients, depending on the affected motor neurons. Muscle paresis of the limbs is seen in spinal-onset disease (>75% of patients), whereas dysarthria is usually the first symptom in patients with bulbar onset.

Amyotrophic lateral sclerosis typically manifests later in life, with a peak incidence in the 7th decade, with males more frequently affected than females (1.5/1; [33]). Death typically occurs within 2–3 years of the onset of first symptoms, mainly due to respiratory failure, but overall survival time ranges from a few months to decades. In Europe, the incidence is about 2–3 per 100,000 individuals [19].

Although defined as a pure motor neuron disease by Charcot in 1869, ALS is now recognized as a multi-systemic disorder that may also affect frontotemporal, oculomotor, cerebellar, and/or sensory systems, and more rarely the basal ganglia and autonomic nervous system [35]. Around 10% of ALS patients fulfill the Neary criteria for frontotemporal dementia (FTD), whereas cognitive impairment with mainly executive dysfunction can be recognized in more than 40% of ALS patients [30]. Initially regarded as distinct diseases, both primary lateral sclerosis (PLS), which affects only upper motor neurons, and progressive muscular atrophy (PMA), which affects only lower motor neurons, are nowadays considered variants of ALS [27].

According to the revised Escorial criteria, diagnosis relies on the identification of upper and lower motor neuron signs in clinical, electrophysiological, and neuropathological examinations, as well as the progressive spread of signs, whereas differential diagnoses are excluded [27]. Treatable differential diagnoses include spinal stenosis, multifocal motor neuropathy, and myasthenia gravis. To date, there is no definitive diagnostic test for ALS, and the clinical diagnosis instead relies on clinical findings, electrophysiological results, and the exclusion of phenocopies. Although not integrated into standard clinical practice, several biomarkers such as cerebrospinal fluid (CSF) neurofilament levels are useful in supporting the diagnosis, particularly in patients with clinically doubtful signs of upper motor neuron involvement [46].

Amyotrophic lateral sclerosis patients should be managed by a multidisciplinary team, including neurologists, psychologists, physiotherapists, pulmonologists, speech specialists, and nutritionists [6]. Symptomatic treatment options include pharmacological and nonpharmacological approaches. For instance, spasticity can be addressed by administration of baclofen whereas hypersalivation can be treated with anticholinergic drugs or Botulinum toxin injection into the parotid glands. Pain, as commonly reported by ALS patients, is treated according to the WHO’s pain relief ladder. Dietary changes (e. g., fluid thickeners) can help to improve nutrition and a gastrostomy tube is an option if severe dysphagia is present. Speech therapy is frequently necessary and assisted communication (customized software) can also be used. Non-invasive ventilation is the preferred treatment for respiratory insufficiency. As substantial immobility and loss of speech are the major problems in advanced disease stages, the patients’ individual wishes for life-prolonging therapies (such as tracheostomy) should be addressed at early disease stages in end-of-life discussions, as cognitive or communication difficulties may arise over time.

In most ALS patients the disease cause is unknown. In up to 25% of cases, however, patients have a family history, with close relatives affected by ALS or FTD. Genetic causes have been identified in sporadic as well as familial cases. This review gives an overview of the most frequently as well as newly identified monogenic causes as well as genetic risk factors of ALS and will discuss ALS-specific aspects of genetic counseling of patients and their families.

## Monogenic causes of ALS

Unraveling the genetic basis of ALS has provided fundamental insights into the pathophysiology of not only familial ALS (FALS), but also of sporadic ALS (SALS). Pathways involved include aberrant RNA metabolism and nucleocytoplasmic transport as well as impaired protein homeostasis. It took until 2011 to identify the most commonly mutated ALS gene, i. e., *C9orf72.* *SOD1*, the second most common and ALS gene to be identified was already linked to ALS in 1993. Both genes together account for around 40% of FALS cases. With the advent of next-generation sequencing techniques, additional disease genes have been found and at present, a genetic cause is found in about 70% of FALS patients and 10% of SALS patients [31]. However, clinically valid frequency data, especially on the more recently identified disease genes, are scarce. The genes most frequently implicated in ALS are inherited in an autosomal-dominant manner with age-dependent penetrance. An overview of all currently known monogenic causes is given in Table 1.

**Table 1:** Monogenic causes of amyotrophic lateral sclerosis (*ALS*) and expected frequencies

Gene	Chromosomal locus	Inheritance	Prevalence familial ALS/sporadic ALS (in percentage)
*C9orf72*	9p21.2	AD, DN	35/5
*SOD1*	21q22.1	AD, AR, DN	15/2
*FUS*	16p11.2	AD, DN	4/1
*TARDBP*/*TDP43*	1p36.22	AD	4/1
*CCNF*	16p13.3	AD	4/2
*NEK1*	4q33	AD	2/2
*TBK1*	12q14.2	AD, DN	1/1
*VCP*	9p13.3	AD, DN	1/1
*SQSTM1*	5q35.3	AD	1/<1
*MATR3*	5q31.2	AD	<1/<1
*CHCHD10*	22q11.23	AD	<1/<1
*PFN1*	17p13.2	AD	<1/<1
*TUBB4A*	2q35	AD	<1/<1
*UBQLN2*	Xp11.21	XL	<1/<1
*OPTN*	10p13	AD	<1/<1
*KIF5A*	12q13.3	AD	NA/NA
*HNRNPA1*	12q13.13	AD, DN	NA/NA
*HNRNPA2B1*	7p15.2	AD	NA/NA
*CHMP2B*	3p11.2	AD	NA/NA
*SETX*	9q34.13	AD	NA/NA
*SPG11*	15q21.1	AR	NA/NA
*ALS2*	2q33.1	AR	NA/NA

*AD* autosomal-dominant, *AR* autosomal-recessive, *DN* de novo, *XL* X-linked, *NA* not available

## Chromosome 9 open reading frame 72

A hexanucleotide (GGGGCC-) repeat expansion in the noncoding region of the gene chromosome 9 open reading frame 72 (*C9orf72*) is the most frequent cause of FALS and FTD. A common genetic cause of ALS and FTD was indeed first proposed in 1991 by linkage analyses, and later genome-wide association studies (GWAS) also pointed to a common underlying genetic factor located in chromosomal region 9p21.2 [24, 39]. Although the exact cut-off between normal alleles and pathogenic expanded alleles is still unclear, repeat expansions with several hundred or thousand repeats are thought to be pathogenic. In European populations, a *C9orf72* repeat expansion was shown to be the underlying genetic cause in up to 35% of FALS and could also be detected in around 5% of SALS patients [50]. *C9orf72*-related diseases include primarily pure ALS, pure FTD or a mixed phenotype of both. *C9orf72* repeat expansions have also rarely been identified in other neurodegenerative diseases, such as Huntington disease-like disorders [20]. Bulbar onset has been more frequently observed in *C9orf72*-related ALS [21].

It is still unclear whether anticipation—as observed in other repeat diseases—also exists in *C9orf72-*associated diseases. Somatic and intergenerational repeat instabilities [21] have been observed: one study reported a repeat expansion of around 70 repeats in a healthy father and an increase to around 1750 repeats in his affected children [45]. Conversely, we observed an expanded allele of 1800–2400 repeats in an affected father and a so-called intermediate allele of 100–120 repeats in the healthy son (unpublished data).

Disease penetrance of *C9orf72*-related ALS is thought to be nearly 100% by the age of 80. No prediction of the individual phenotype, i. e., ALS, FTD or ALS/FTD, the exact age at onset, the disease course, and disease duration is currently possible.

To detect the *C9orf72* repeat expansion, PCR-based amplicon fragment analyses, repeat-primed PCR protocols, and Southern blotting are used. The gold standard for detecting the *C9orf72* repeat expansion is Southern blotting, as PCR-based techniques are still failure-prone [1].

The molecular mechanisms underlying neurodegeneration in *C9orf72*-related diseases are a matter of debate and several different, nonmutually exclusive pathomechanisms have been described. The *C9orf72* repeat expansion may be associated with aberrant RNA metabolism because of sequestration of RNA binding proteins, production of abnormal RNA species, or increasing DNA instability due to the formation of RNA–DNA hybrid structures [42]. Altered protein homeostasis may result from impaired autophagy or accumulation of dipeptide repeat proteins after non-ATG mediated translation [25]. Dipeptide repeat proteins have also been shown to interfere with nucleocytoplasmic transport [48]. Last but not least, haploinsufficiency could be another explanation [34].

## Superoxide dismutase 1

The superoxide dismutase 1 (SOD1) protein is ubiquitously expressed. It encodes a homodimeric enzyme that catalyzes the reduction of superoxide anions (a reactive oxygen species) to oxygen and hydrogen peroxide. Most *SOD1* mutations reported to date are missense mutations, but smaller deletions and duplications have also been detected [4]. Mutations in *SOD1* are found in around 15% of FALS patients and up to 2% of SALS patients [50]. Most mutations in *SOD1* have been identified in families with autosomal-dominant ALS. However, the common mutation D90A (according to HGVS c.272A>C p.Asp91Ala, NM_000454) can also be inherited in an autosomal-recessive manner [5]. Recently, emerging evidence suggests that neuronal aggregates of misfolded SOD1 protein might have prion-like properties and cause a fulminant ALS-like phenotype when injected intraspinally in minute amounts into 100-day-old healthy mice [7].

## TAR DNA binding protein and FUSED IN SARCOMA

Accumulation of proteinaceous inclusions in motor neurons are a neuropathological hallmark of ALS and TDP43-postive inclusions can be detected in around 95% of all ALS patients [26]. However, *TDP43* mutations can be identified in only a few patients. *TDP43* mutations account for around 4% of FALS and 1% of SALS cases [50]. Most mutations published to date (Human Gene Mutation Database, HGMD, *n* = 44) are missense mutations and are mainly located within the C‑terminal part of the protein. TAR DNA-binding protein 43 (TDP43) as well as FUSED IN SARCOMA (FUS) are RNA-binding proteins and have been shown to mislocalize from the nuclear to the cytoplasmic compartment when mutated. A loss of normal processing of their target RNA is one of the hypothesized pathomechanisms [3, 49]. Both proteins also contain prion-like domains, which may also represent a disease mechanism. Mutations in *FUS* can be identified in around 5% of FALS and 0.5% of SALS patients [50]. Of note, *FUS *mutation frequency is especially high in sporadic, early onset (<35 years of age) ALS patients because of de novo mutations [22]. On the contrary, there are no convincing data concerning a major contribution of de novo mutations in additional genes in ALS pathogenesis [16], although this has been claimed by initial studies [12, 37].

## Novel disease genes

Within the last 5 years, nine novel genes associated with monogenic forms of ALS have been identified: *KIF5A*, *CCNF*, *NEK1*, *TBK1*, *MATR3*, *TUBA4A*, *CHCHD10*, *HNRNPA1*, and *HNRNPA2B1* [10, 13, 43]. Frequency data on mutations in these genes are scarce and rough estimates exist for only eight of these genes (*KIF5A*, *CCNF*, *NEK1*, *TBK1*, *MATR3*, *TUBA4A*, *CHCHD10*).

Missense mutations in *KIF5A*’s N‑terminal motor domain or coiled-coil domain and heterozygous de novo frame-shift mutations in its C‑terminal part were associated with hereditary spastic paraplegia 10, Charcot–Marie–Tooth disease type 2 (CMT2), and neonatal intractable myoclonus respectively. Very recently, *KIF5A* was also implicated in ALS [10]. Two splice-site mutations and a rare missense mutation, leading to loss of mutant RNA or aberrant splicing, were identified in FALS patients, segregating with the phenotype within the families. Furthermore, a single non-synonymous SNP (rs113247976, c.2957C>T p.Pro986Leu) was significantly enriched in FALS patients (3.40% vs 1% in controls *P* = 1.28 × 10^−7^; [10]).

Mutations in *CCNF* were identified in both ALS and FTD, and are thought to account for 4% of FALS and 2% of SALS cases [43]. *NEK1 *mutations were associated with ALS without dementia and found in up to 2% of ALS cases [9]. By burden analysis, nonsynonymous variants in *TBK1* were found to be enriched in ALS patients, but the number of *TBK1*-related ALS is low (around 1% of FALS and 1% of SALS cases; [15, 18]). Even less frequently, mutations in *MATR3*, *TUBA4A*, and *CHCHD10* can be detected [13].

The proteins encoded by these recently identified disease genes are involved in several intracellular pathways known to be implicated in ALS or interact with known ALS genes. *MATR3* was shown to interact with FUS and TDP43 and regulate gene expression [23, 47]. *CHCHD10* is localized to the mitochondrial intermembraneous space and is important for mitochondrial maintenance. Some data suggest that *CHCHD10* pathology is caused by haploinsufficiency [11]. *CHCHD10* also interacts with TDP43 and promotes its retention to the nucleus [44]. Missense mutations in *TUBA4A* prevent the encoded alpha tubulin from polymerizing and result in a disturbed microtubule network [36]. *TBK1* interacts with several proteins and is involved in cellular processes such as autophagy, neuroinflammation, and ubiquitin-mediated protein degradation [18]. Also, *CCNF* was shown to be part of the ubiquitin-dependent protein degradation processes [43].

## Genetic risk factors

Amyotrophic lateral sclerosis is a genetically heterogeneous disorder with monogenic as well as multifactorial inheritance, and boundaries are fluid. Some genetic risk factors for ALS have been identified by genome-wide association studies (GWAS). It is suggested that around 8.5% of heritability of ALS is tagged by common SNPs. However, only 0.2% of variance in liability can currently be explained by six common susceptibility loci in/near the genes *UNC13A*, *SARM1*, *MOBP*, *SCFD1,* *C21orf2*, and* C9orf72 *[32]. For the latter, several SNPs have been shown to contribute to a disease haplotype that influences the length of the hexanucleotide repeat [29]. In the future, increasing sample sizes may help to unravel more SNP-based heritability in ALS.

TDP43 toxicity was shown to be increased in yeast by expression of Ataxin 2 (*ATXN2*). Indeed, in humans, intermediate-length polyglutamine stretches (27–33 glutamines) confer a risk for ALS, whereas repeat stretches larger than 34 glutamine repeat are associated with spinocerebellar ataxia type 2 [17]. With the exception of research projects, genetic testing of (complex) genetic risk factors is of minor clinical relevance to date.

## Genetic testing and counseling

The diagnosis of ALS is based on clinical and neurophysiological findings. With new sequencing technologies, clinical genetic testing becomes more feasible and offers the possibility of a definitive diagnosis in a growing number of cases [40]. The probability of identifying a genetic cause is higher in familial than in sporadic ALS. Indeed, a genetic cause in seemingly sporadic ALS can be masked by recessive inheritance, reduced penetrance, small family size, lack of family information, and illegitimacy. Within the last few years, neurologists started offering genetic counseling to ALS patients more frequently and patients who underwent genetic counseling reported a positive experience and found value in testing [41]. According to the guidelines of the German Society of Neurology, genetic testing should be offered to patients with familial ALS, patients who have family members with dementia, and patients with an early onset and rapid progression [28]. More recent recommendations suggested diagnostic *C9orf72* testing in all ALS patients, regardless of family history [14].

In a first diagnostic step, i. e., before multigene sequencing approaches are applied, a repeat expansion in *C9orf72* as the most frequent genetic cause of ALS should be excluded. The genes tested in custom-made panels vary depending on the laboratory. In a routine clinical setting, we suggest that at least the most frequent disease genes, such as *SOD1*, *FUS*, and *TPD43*, should be tested in all patients. For research purposes and maybe also in clinical settings, further genes may be analyzed. But especially for recently identified genes, variant interpretation may be challenging. Obviously, the identification of variants of unknown significance is more probable in newer disease genes.

In around 5% of families with FALS, a pathogenic mutation in more than one ALS gene could be detected, providing evidence for oligogenic inheritance in ALS [8].

Owing to the absence of preventive medical treatment and the natural disease course of ALS, predictive genetic testing is challenging and shares similarities with other neurodegenerative disorders, such as Huntington disease. Variable expressivity, age-dependent penetrance, and oligogenic inheritance further complicate genetic counseling in healthy relatives who seek to know about their disease risks. In a survey of neurologists specialized in ALS, only 52% stated they offer predictive testing to ALS relatives and one-fifth of neurologists who would offer predictive testing would not recommend it to their own family [38]. However, formal provision of genetic testing for those who have a first- or second-degree relative with ALS is recommended [14]. Given the aforementioned complexities, one might argue against predictive testing in ALS. But for the individual case, there may be several personal reasons why one might opt for predictive testing, such as life decisions like going on a round-the-world trip with a caravan before or after retirement (as reported by a 57-year old woman whose sisters both carried a *SOD1* mutation and developed ALS by the age of 63 and 61 years respectively). Younger individuals may choose not to have children or ask for preimplantation genetic testing. Most people express that the anxiety of living without knowing is worse than living with the knowledge. In our experience, a clear communication of the genetic complexity in ALS is of utmost importance for people undergoing predictive testing. It must be clearly mentioned that genetic testing only addresses the genetic alteration already documented in the family and does not test all known genetic variants in ALS. Issues linked to variable expressivity and age-dependent penetrance should be communicated. Similar to predictive testing in HD, multiple counseling sessions including predecision, pre-test, and post-test counseling sessions are desirable. Additional counseling sessions should be offered if necessary. Obviously, the decision to undergo testing should be voluntary, and informed consent as well as psychological readiness (exclusion of active psychiatric conditions) are prerequisites. Consulters may decide at any time not to receive or to delay receiving the test results. Detailed recommendations for predictive testing of ALS were recently published [14].

## Outlook

The knowledge of the biological and genetic basis of ALS and the advances in the care of ALS patients have improved substantially within the last few years. Genetic causes of ALS have been identified in both sporadic and familial patients and the number of disease genes involved is still increasing. One might guess that most of the monogenic forms are already identified, but there is still “missing heritability,” which might be explained by rare variants with large effect sizes. The identification of genetic causes of ALS will help to develop new therapeutic approaches, either by the identification of shared disease pathways such as TDP43 pathology or by targeted therapies for known mutations. Currently, antisense oligonucleotide trials in *SOD1*- and *C9orf72*-related ALS are being conducted. Besides riluzole, the second modifying medication, edaravone, became FDA-approved last year. Whether it is beneficial to all ALS patients or just to subgroups needs to be evaluated within the next few years [2]. In the future, biomarkers will hopefully help to monitor disease progression and genomics or transcriptomics will help to further personalize treatment to one’s individual disease subtype.
